# Fulminant myocarditis parvovirus B19 related in a young woman

**DOI:** 10.1007/s10047-021-01247-7

**Published:** 2021-01-24

**Authors:** Rita Pesce, PierPaolo Taffarello, Stefania Rizzo, Cristina Basso, Luisa Cacciavillani, Alvise Del Monte, Luciano Babuin, Gino Gerosa, Tomaso Bottio

**Affiliations:** 1 Cardiosurgery Unit, Department of Cardiac-Thorac-Vascular Sciences and Public Health, Via Giustiniani 2, 36100 Padua, Italy; 2 Cardiovascular Pathology Unit, Department of Cardiac-Thorac-Vascular Sciences and Public Health, Padua, Italy; 3 Cardiological Unit, Department of Cardiac-Thorac-Vascular Sciences and Public Health, Padua, Italy

**Keywords:** Parvovirus PB19, Fulminant lymphocytic myocarditis, Rescue therapy, LVAD

## Abstract

We present the case of a 18-year-old female with fulminant lymphocytic myocarditis caused by Parvovirus B19 (PVB19), successfully treated using temporary LVAD. In the literature there is no consensus on the surgical strategy. While some surgeons prefer to use a single device supporting only the LV, others prefer to start immediately with a biventricular supporting. At pre-procedural ultrasound evaluation, her anatomical features were not suitable for a percutaneous device such as the Impella. Thus, a temporary paracorporeal continuous flow LVAD was inserted. The heart recovery allowed LVAD removal 9 days after the implant.

**Supplementary Information:**

The online version contains supplementary material available at 10.1007/s10047-021-01247-7.

## Introduction

Acute myocarditis has various aetiologies, and clinical presentation and specific treatments could be very different [1]. We present a case of fulminant lymphocytic myocarditis caused by Parvovirus B19 (PVB19) infection, treated successfully by temporary left ventricle assist device (LVAD) implantation.

## Case presentation

**Fig. 1:**
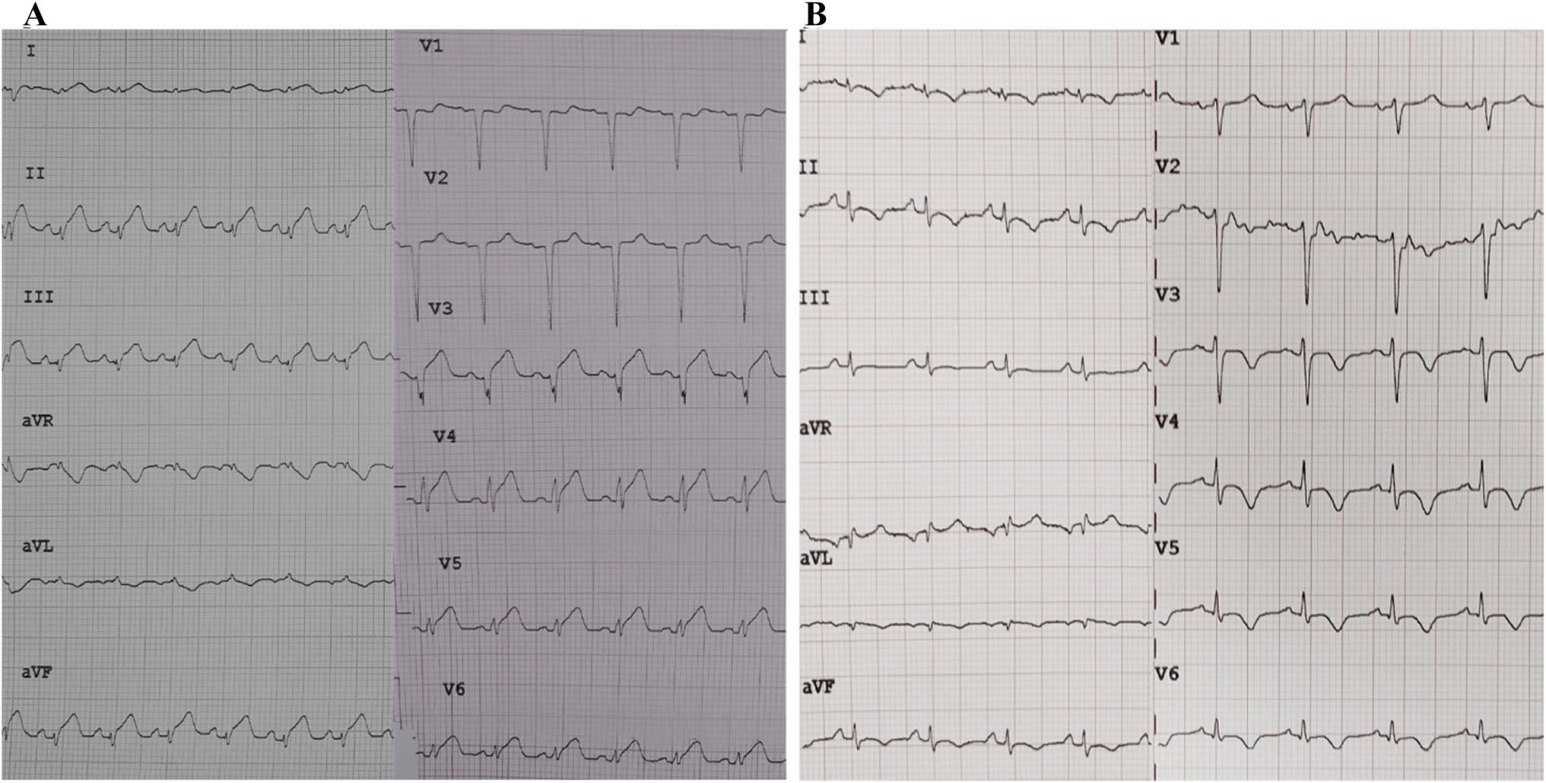
**a** First ECG: general ST segment elevations; **b** Last ECG: no more ST segment elevations evident

**Fig. 2:**
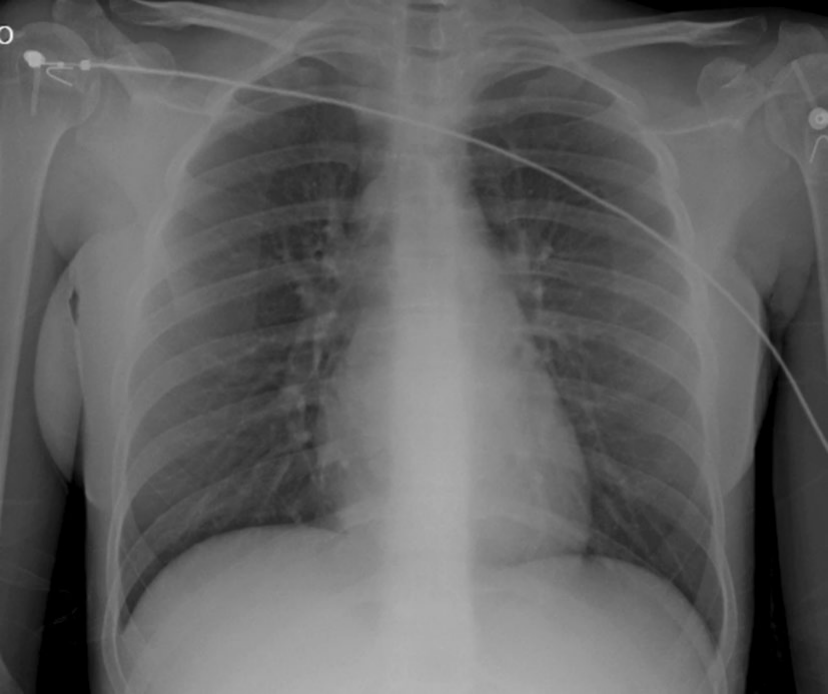
Chest X-ray at admission

The ethical committee approved the study [IRB number 62813 (10/21/2020)]. A female patient, 18 years, without cardiovascular risk factors, presented with cough, high fever, chest pain and dyspnoea. The ECG is shown in Fig. 1. A severe biventricular dysfunction was evident (TTE) (video 1). WBC (16.17 × 10^9^/L), ALT (989 U/L) AST (1113 U/L), total bilirubin (20.2 umol/L), CPK (2549 U/L) and troponin I (9701 ng/L) were higher than normal. Spontaneous INR was 1.85. The nasal swab was positive for N1-H1 viruses. Blood gas analysis was acidotic with pH 7.19. Chest x-ray was abnormal (Fig. 2).

After a first medical attempt to stabilize the patient, the clinical case was discussed in Heart Team. The size of the femoral and axillary arteries was too small precluding the use of an Impella. A surgical approach was chosen.

At the anaesthesia induction, the patient had a cardio-circulatory arrest. Soon after resuscitation and temporary pharmacological stabilization, we implanted the paracorporeal LVAD (Levitronix pump GmbH), with interposed a membrane oxygenator (Thoratec Corporation, USA), performing very quickly the procedure off pump. The inflow cannula was inserted in the left ventricle through the left ventricle apex, through a small left mini-thoracotomy (3–4 cm in length). The cannula (Single Stage Venous Cannula, Medtronic, 28 Fr) was inserted through the “sew-then-core” technique. We usually fix the sewing cuff to the heart with 4 interrupted polypropylene pledgeted mattress sutures. Subsequently, an eleven blade scalpel is used to core the myocardium to permit a blunt cannula insertion. The mattress sutures have been placed with epicardial stitching. Epicardial stitching of the sewing ring provides the best results to prevent suction events as well as thrombosis formation. The external part of the cannula is then fixed to the skin with transdermal stitches. The correct placement is confirmed by intraoperative transesophageal echocardiography (TEE).

**Fig. 3:**
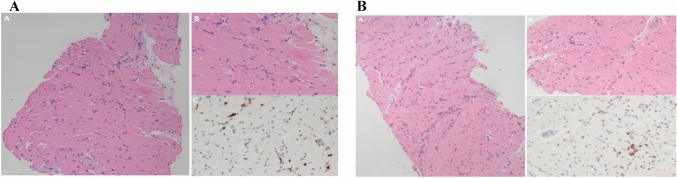
**a** Plurifocal inflammatory infiltrate with myocyte necrosis, consistent with acute lymphocytic myocarditis (**a**, **b** hematoxylin and eosin, scale bar 100 micron and 50 micron). Immunostaining for leukocyte marker CD3 showed a number of T lymphocytes > 7/mm^2^ (scale bar 50 micron). **b** Focal inflammatory infiltrate with myocyte necrosis, consistent with persistent lymphocytic myocarditis (**a**, **b** hematoxylin and eosin, scale bar 100 micron and 50 micron). Immunostaining for leukocyte marker CD3 showed a focus of T lymphocytes < 7/mm^2^ (scale bar 50 micron)

The outflow cannula was inserted in the left common femoral artery, through a small groin incision. The femoral vessels had a maximum diameter of 5.5 mm and we inserted 15 French arterial cannula (OptiSite, Edwards Lifesciences). The left femoral artery inflow cannula was placed percutaneously in Seldinger technique, especially considering the emergent situation and being the fastest method in the experienced surgeon’s hands. Thereafter, the external part was fixed with transdermal stitches to the patient’s leg. Peripheral perfusion was accomplished through an additional superficial femoral artery cannulation (Avanti + , Cordis 7 French). A myocardial biopsy was performed. An active lymphocytic myocarditis due to a PVB19 virus infection with a viral load > 500 genome equivalents (GE)/µg was the final diagnosis (Fig. 3a).

**Fig. 4:**
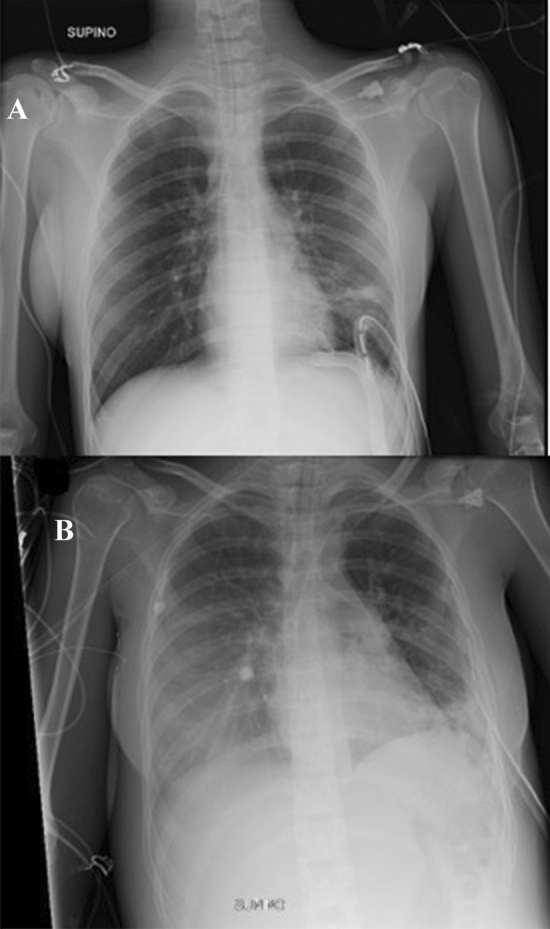
Chest X-ray soon after LVAD insertion (**a**) and after LVAD removal (**b**)

The procedure occurred without complications. The TEE showed a mild right ventricle (RV) depression, but enough to fill the LV, thus justifying the single LVAD (blood flow of 3.35 L/min; total theorical blood flow: 3.45 L/min). Patient was extubated within few hours. The reason we want early extubation is to have the autonomous catecholaminergic contribution.We started oseltamivir 75 mg twice immediately for N1-H1 positivity. Lactate levels were normalized within 12 post-operative hours. TnI level had a post-operative peak of 33,011 ng/L, but after 5 days it became negative (lower than 34 ng/L). Since the second post-operative day, we started to see a minimal flow pulsatility. The first TTE (third post-op day) showed normal diameters with a diffused hypokinaesia. A RV moderate dysfunction was evident (TAPSE 0.60 cm, SF 19%). Day by day we observed an increase in flow pulsatility. Soon after LVAD pump on, the achieved blood flow was 3.35 L/min (total theorical blood flow: 3.45 L/min). Furthermore, the hemodynamic was supported by pharmacological infusion of dobutamine (6 μg/kg/min), adrenaline (0.08 μg/kg/min) and noradrenaline (0.08 μg/kg/min). About three or 4 h after surgery, in the absence of bleeding, we start infusion of heparin to obtain a PTT of 40–50 s. From the day after, we witnessed an improvement in the patient's general conditions: decreasing of lactate levels, improvement in lung function to allow extubation in a short time, warm and well perfused limbs, a rapid reduction of AST, ALT and INR levels and a slight biventricular function improvement in TTEs performed on the first, third, fifth and eighth day after implantation. The hemodynamic remained stable with MAP values around 70–80 mmHg, despite the considerable reduction in LVAD blood flow day after day (3 L/min the 2nd day after; 2.25 L/min the 4th day after; 1.2 L/min the 6th day after; 0.5 the 8th day after). Day by day we observed an increase in flow pulsatility. The LVAD has been successfully weaned 9 days after the implant, leaving a minimal inotropic support of dobutamine and adrenaline finally discontinued (Fig. 4a, b). Subsequently, the pharmacological weaning took place in a gradual manner without complications, maintaining stable hemodynamics (PAS > 90 mmHg).

The extubation was possible few hours after surgery. Control TTE is shown in video 1. The ECG at discharge was the following (Fig. 1b). A second biopsy was performed (Fig. 3b). The day after, the patient was discharged from the ICU.

## Discussion

Myocarditis, an inflammatory disease of the myocardium, is a challenging diagnosis due to the heterogeneity of clinical presentations. Endomyocardial biopsy is the gold standard for the diagnosis [1]. The histological diagnosis of myocarditis includes different forms: lymphocytic, eosinophilic, polymorphic, giant cell myocarditis, and cardiac sarcoidosis [1]. Acute myocarditis resolves in about 50% of cases in the first 2–4 weeks, but 12–25% may acutely deteriorate and either die or progress to end-stage dilated cardiomyopathy [2]. In unstable conditions, supportive care includes vasopressors, positive inotropic agents, diuretics, vasodilators and VADs [2]. In the literature, cases of Parvovirus B19 correlated myocarditis has been described rarely, and the clinical presentation was less severe than in our case [3]. Although the pathogenetic role of PVB19 genomes in myocarditis has not yet been sufficiently elucidated, Bock et al. suggested that PVB19 viral loads of more than 500 ge per microgram in endomyocardial biopsy specimens are a clinically relevant threshold for the maintenance of myocardial inflammation [4]. Moreover, recently, PVB19 was the only detected virus in all molecular positive cases of investigated lymphocytic fulminant myocarditis [5]. In case of acute myocarditis, the surgical implantation of a paracorporeal LVAD is an effective therapeutic strategy, and while performed minimally invasive it is even safer than through a median sternotomy for the following reasons. In case of sternotomy the hemodynamic instability limits the exposure of the LV apex, thus requiring the use of CPB or temporary ECMO and the subsequent use of heparin which might compromise the coagulation pathway. On the other hand, a minimally invasive approach through a left mini-thoracotomy, does not force dislocation of the heart (the apex remains right in front of the surgeon), avoiding the need of CPB, the full heparin dose, therefore, limiting the bleeding risks.

Even though, results of VA-ECMO are excellent [6], when the self LV venting is un-sufficient for poor residual contractility, the patients should be assisted with an LVAD with the aim to avoid pulmonary oedema, cardiac chambers thrombosis, increased left ventricular wall stress and delayed contractility recover.

In the literature there is no consensus on the surgical strategy. While some surgeons prefer to use a single and/or double device support for the LV and RV, according to the severity of the symptoms and gravity of the score related to renal, hepatic and respiratory dysfunctions, others prefer to start immediately with a BiVAD support [7].

Two are the concepts that we would like to share. First is the method we used: step by step approach demonstrates that it is possible to avoid a BiVAD support even in biventricular acute failure. The patient’s liver function had a marked improvement already after about 48 h from the LVAD implantation: the INR value at the entrance was 2.85, the 2nd day after was 2.50, the 3rd day after was 1.84, the 5th day after was 1.4 and it remained stable until discharge. At the hospitalization, ALT value was 1132 U/L, we have a peak value of 2790 U/L after 24 h, but after a few hours the levels began to decrease constantly, until it reached a value of 290 U/L on weaning day. At the beginning, AST value was 1321 U/L, but after just 24 h it began its gradual decrease, in conjunction with the reduction of ALT levels, up to a value of 175 U/L on weaning day. In addition, the total bilirubin level was high at the hospitalization (20.2 umol/L) and after 24 h returned into the normal range; its value was 7.1 umol/L on the weaning day. We carefully monitored all the clinical and echocardiographic signs that could indicate a RHF, but fortunately none of them have occurred. Generally, post-LVAD RHF is suspected in the presence of low-pump output with signs of low cardiac output (low MAP, decreased urine output, increasing lactate levels) and of congestive heart failure (increased CVP, worsening hepatic function, peripheral oedema, ascites). Definitive diagnosis is made by TEE/TTE. Echo signs of RHF include: dilated RV (the various postoperative TTE performed have never found a right ventricle dilation), qualitatively reduced RV systolic function, shifting of the interventricular septum toward the LV, reduced or virtual LV cavity, new or worsened tricuspid regurgitation, dilated non-collapsing IVC. Among all these specific signs, we noticed just a reduced RV systolic function with TAPSE 0.60 cm and SF 19% at the 3rd postoperative day, but we noticed an improvement of the SF (19–27%) at the 5th postoperative day. Only when in presence of a persistent postoperative low output state with all the described symptoms and clinical signs, a temporary RVAD with a BiVAD configuration should be taken into consideration. In these cases, a new centrifugal pump is generally used and the femoral vein is cannulated in the groin with a two-stage cannula (Maquet Ltd) and the pulmonary artery with a percutaneous arterial cannula with Seldinger technique (Maquet Ltd), through a mini thoracotomy in the second left intercostal space.

Second, is that although the inflammatory process is equivalent in the two biopsies, in fact the last biopsy has not changed as regards the inflammatory infiltrate, what has changed is the dramatic reduction of the viral load at the immunohistochemical investigation, confirming the viral load itself as the critical point.

## Conclusion

In this particular case, we demonstrated the efficacy of isolated LVAD implant to treat PVB19 fulminant myocarditis in an emergency setting.

## Supplementary Information

Below is the link to the electronic supplementary material.

Supplementary file1 Video 1: Pre-op: Severe biventricular dysfunction, oedema of left ventricular walls, hyper-reflective myocardial areas, no ventricular thrombi, moderate pericardial effusion; Post-op: normal biventricular contractility, no more evidence of hyper-reflective myocardial areas, left ventricular “oedema” partially reverted. (MOV 22356 KB)
